# Knowledge of Emergency Physicians about the Management of Traumatic Dental Injuries: A Cross-sectional Study in Northern Iran

**DOI:** 10.4314/ejhs.v32i2.16

**Published:** 2022-03

**Authors:** Touraj Assadi, Mahmood Moosazadeh, Pouya Akbari, Salma Omidi

**Affiliations:** 1 Department of Emergency Medicine, Faculty of Medicine, Mazandaran University of Medical Sciences, Sari, Iran; 2 Health Sciences Research Center, Addiction Institute, Mazandaran University of Medical Sciences, Sari, Iran; 3 Faculty of Dentistry, Mazandaran University of Medical Sciences, Sari, Iran; 4 Dental Research Center, Mazandaran University of Medical Sciences, Sari, Iran; 5 Department of Endodontics, Faculty of Dentistry, Mazandaran University of Medical Sciences, Sari, Iran

**Keywords:** Knowledge, Dental Occlusion, Traumatic, Emergency Service, Hospital, Physicians

## Abstract

**Background:**

Dental trauma is a common dental public health problem among the children and adults. As the emergency departments are usually the first contact point for patients after a trauma, this study aimed to evaluate the level of knowledge of emergency medicine specialists or residents and general practitioners (GPs) working in the emergency departments about the management of traumatic dental injuries.

**Methods:**

In this descriptive cross-sectional study, all emergency medicine specialists or residents and GPs working in the emergency department of hospitals affiliated to Mazandaran University of Medical Sciences were evaluated. Data were collected using a questionnaire comprising questions on demographic information and physicians' knowledge of how to deal and manage different dental injuries. The information was analyzed using t-test and chi-square statistical test.

**Results:**

The level of knowledge was good in 71.5% of emergency medicine specialists and average in 75% of the residents and 93.4% of GPs. None of the participants had low level of knowledge about management of traumatic dental injuries. There was a statistically significant association between physicians' knowledge and their job position (P<0.001). Moreover, there were no statistically significant differences between gender and the history of participating in dental trauma courses with the level of physicians' knowledge (P>0.05).

**Conclusion:**

The results of present study highlights the need for training programs to raise the knowledge of emergency medicine residents and GPs about how to effectively manage the traumatic dental injuries in patients presenting to the emergency department.

## Introduction

Dental trauma is common among children and adults that may affect teeth, hard dental tissues, and dental support structures (1–2). Falling down, injuries from cycling and other sports, injuries from to car accidents, as well as certain diseases like epilepsy are known as etiological factors of dental trauma (2–6). Besides leading dental and supporting tissues lesions, oral traumatic injury directly or indirectly impacts people's lives by affecting people's appearance, speech, and tooth position (7, 8). Additionally, it is important to note that traumatic dental injuries can cause functional, aesthetic, psychological and social problems and have a significant effect on quality of life in individuals with traumatic dental injuries (9–11).

According to pre-defined classification, dental injuries are divided into two general classes as fractures and dislocations. Dislocations include concussion, subluxation, extrusion, lateral luxation, and intrusion. Moreover, complete displacement of a tooth from its socket in alveolar bone is called avulsion (1, 12). The prognosis of a tooth following trauma is critical. This depends on various factors such as the severity of the injury, the quality and timing of initial treatment, and appropriate follow-up and care (1, 13–15). It has been previously confirmed that early appropriate management of dental trauma is crucial in the outcome and prognosis of injured tooth (12, 15). Thus, the initial intervention and management at the moments, just after the trauma to the tooth, could have a great effect on the final prognosis of the tooth. For instance, the treatment of an avulsed tooth differs depending on how long it has been out of socket and what the developmental stage of the apex is. In this type of injuries, early and rapid intervention and the maintenance of the avulsed tooth have a drastic effect on the final success of the treatment (16–17).

It is generally accepted that all traumatic dental injuries have to be considered and treated as an emergency. Emergency departments of hospitals are usually the first contact point for patients after a trauma (18–20). Usually a dentist is not available in emergency departments. Therefore, appropriate familiarity of emergency physicians with the management of traumatic dental injuries is crucial. Physicians working in the emergency department of hospitals who are at the front line of trauma management include emergency medicine specialists, emergency medicine residents, and general practitioners (GPs). They usually face complex types of trauma in their work environment, Head and neck trauma is of these cases that can be accompanied by dental injuries. Moreover, a patient with a single dental trauma can visit the hospital as well. This is while, some studies reported that most of the emergency physicians are not sufficiently aware of the management of traumatic dental injuries (21–23). In addition, it has been previously shown that a large proportion of emergency department physicians do not receive any formal education about management of traumatic dental injuries (5, 18). As the emergency departments of hospitals are generally the first contact point for people with a trauma, and given the importance of sufficient knowledge of emergency physicians for effective management of dental trauma and the fact that no studies have been conducted in this regard in Iran, the aim of this study was to evaluate the knowledge of emergency medicine specialists and residents and GPs working in the emergency departments about the management of traumatic dental injuries.

## Methods

In a descriptive cross-sectional study a total of 41 emergency medicine specialists and residents and also GPs working in the emergency departments of educational hospitals affiliated to Mazandaran University of Medical Sciences in 2018, were included. Given the limited number of GPs, emergency medicine specialists and residents, all of them entered the study using a census method. For data collection, a researchermade questionnaire, whose validity and reliability have been proven by literature review and expert opinions, was used. A total of 43 self-administered questionnaires were distributed and the physician's participation was voluntarily. Two physicians refused to participate and complete their questionnaires because they were exhausted or busy. For content validity, the questionnaire was confirmed by ten professors of dentistry and emergency medicine. Cronbach's alpha was used to determine the internal consistency reliability of the questionnaire (α>0.8). The questionnaires were distributed to participants. Each questionnaire consisted 15 questions in two parts. The first part had 7 questions in terms of sociodemographic characteristics including gender, age, graduation year, and whether they took a course specifically about dental trauma. The second part had 10 questions regarding the knowledge of traumatic dental injuries management in the following aspects: how to deal with teeth with lateral luxation and intrusion, the significance of immediate management of permanent avulsed teeth in prolonged success in treatment, the importance of not replacing deciduous teeth given the potential risk of damage to permanent teeth, the critical time for teeth being out of the alveolar bone, avulsed tooth care procedures and extremely contaminated tooth cleaning technique prior to placement.

A score of 1 point for a correct answer and 0 for wrong answer was given for the 10 questions in second part. Scores 7-10 are considered good, scores between 3 and 6 are considered average, while scores between 0 and 2 were considered as weak knowledge of participants (24).

**Ethical consideration**: This study was approved by the ethics committee of Mazandaran University of Medical Sciences (code: IR.MAZUMS.REC.1397.3247). Explanation regarding the aim of this study was given to all the participants and they were assured that the study would be conducted anonymously and confidentially and their participation in the study is completely voluntary. Answering and returning the questionnaire was considered informed consent, and all participants were informed in writing that the reported data would be published.

**Sample size calculation**: Estimation of sample size was based on previous study (20). At a level of α=.05 with a power of 0.9, we calculated that it was necessary to enroll 38 eligible participants for this study, so in order to allow for a withdrawal, we planned to recruit 41 participants.

**Statistical analysis**: Data were analyzed using Statistical Package for the Social Sciences (SPSS) software (version 16.0, SPSS Inc., Chicago, IL, USA). In this study, descriptive statistics (number, percent, mean, standard deviation, minimum and maximum) and inferential statistics (Chi-square and T-test) were used. P-value <0.05 was considered statistically significant.

## Results

Forty one physicians participated in the study, 15 GPs, 12 emergency medicine residents, and 14 emergency medicine specialists. Among them, 26 were males (63.4%), and their mean age was 42.96±8.3 years.

Nine of the participants took a course in management of dental trauma, and 27 of them (65.9%) had experience of managing patients with dental trauma. Table 1 shows the number of correct responses of the participants to the questions. Overall, 61.2±1.64% of participants correctly answered to the questions. Level of knowledge of participants with different job position are presented in Table 2. None of the participants had weak knowledge about the management of traumatic dental injuries.

**Table 1:** The number of correct responses of the physician for each question

Questions	Number (%) of correct responses
How to deal with lateral displacement of two permanent central teeth	26 (63.4)
How to deal with evulsion of permanent central teeth in a 7-year-old child	24 (58.5)
How to deal with the complete avulsion of a permanent tooth in the past one hour	18 (43.9)
How to deal with a complete permanent tooth avulsion in the last 6 hours	17 (41.5)
How to deal with an infected tooth before replacing it	38 (92.6)
Suitable environment for keeping the avulsed tooth	12 (29.3)
The best time to replace the avulsed tooth	31 (75.6)
Which part should be an avulsed tooth held from?	26 (63.4)
The age of growth of permanent maxillary central teeth	25 (61.0)
The need to replace the avulsed deciduous teeth	32 (78.0)

**Table 2:** Level of knowledge of all physicians based on their job position

Job Position	Parameters	Average	Good	Total
**GPs**	Frequency	14	1	15
	Percentage in field	93.4%	6.6%	100%
**Resident of emergency medicine**	Frequency	9	3	12
	Percentage in field	75%	35%	100%
**Emergency medicine specialist**	Frequency	4	10	14
	Percentage in field	28.5%	71.5%	100%
**Total**	Frequency	27	14	41
	Percentage in field	65.9%	34.1%	100%

There was a statistically significant difference between the physicians' knowledge and their job position (*P*<0.001), so that emergency medicine specialists had a better level of knowledge than emergency medicine residents and GPs. Table 3 shows the knowledge of all physicians by gender, where no statistically significant differences were found between gender and level of knowledge (*P*=0.339). Table 4 shows the level of knowledge of physicians by history of taking a course in management of dental trauma. Also, there was no significant statistical differences between the history of taking a course in management of dental trauma and level of participants' knowledge (*P*=0.129). The mean age of physicians with average and good knowledge was 43.14±9.43 and 42.42±9.34 years, respectively (*P*=0.863).

**Table 3:** Level of knowledge of all physicians by gender

Gender	Parameter	Average	Good	Total
**Male**	Frequency	16	10	26
	Percentage in field	61.6%	38.4%	100%
**Female**	Frequency	11	4	15
	Percentage in field	73.4%	26.6%	100%

**Table 4:** Physicians' level of knowledge by history of taking a course in management of dental trauma

History of dental trauma course	Parameter	Average	Good	Total
**Yes**	Frequency	23	9	32
	Percentage in field	71.9%	28.1%	100%
**No**	Frequency	4	5	9
	Percentage in field	44.4%	55.6%	100%

## Discussion

Epidemiological studies indicate that the incidence of dental trauma varies drastically, most probably due to different parameters such as nationality, age and gender of patients as well as different classification systems being used for dental trauma in the published studies. Thus, their results cannot be easily compared (1–2, 4, 25). A recent systematic review of dental trauma showed that one-third of all pre-school children have trauma to deciduous teeth, and one-fourth of all school-age children and almost one-third of adults suffer from trauma to permanent teeth, but changes varies among and within different countries (26).

The questionnaire used in this study consisted of 10 questions where the participants' information about dental trauma was evaluated. A question was about the management of a patient with lateral luxation and the correct response was to replant the tooth if possible and then visiting a dentist, with 63.4% of the participants responding correctly. Another question was about the patient with intrusion. The correct response was a quick visit to the dentist, with 58.5% responding correctly. The next question was about the management of a patient with evulsion in less than an hour, with the correct response having the tooth replanted if possible and then visiting a dentist. Less than half of the participants responding correctly to this question. The fourth question was asked about the same issue, except that its avulsion time was more than 6 hours, where again less than half of the participants responded correctly. The possible explanation for this low level of knowledge can be that patients who presented to the emergency departments usually have multiple traumas, and medical conditions that threaten the patient's life are generally considered.

The lowest number of correct responses was related to the eighth (which part of the avulsed tooth should be kept) and the sixth questions (the best storage environment for the avulsed tooth). The correct responses to these questions were from the crown of the tooth and keeping it in the milk. It may be due to the more specialized nature of these questions in the field of medicine and the different teaching resources of medicine and dentistry. The seventh question was about the best time to replace an avulsed tooth, with 75% choosing the correct response as “quickly or within half an hour.” The eighth question was where the extracted tooth should be kept, with 63% choosing the correct response. The ninth question was about the growth time of permanent central tooth, with 61% choosing the right time. The last question was about replacing the deciduous teeth, with 78% stating no need to replace deciduous teeth.

The results of this study indicated that the mean level of knowledge of emergency physician was average. Emergency medicine specialists had significantly higher levels of knowledge than emergency medicine residents and GPs. There was no statistically significant relationship between gender, history of dental trauma course, and experience of managing patients with dental trauma with level of knowledge of emergency physicians. As the level of knowledge between emergency medicine residents was somewhat different from each other, one can infer that the year and semester of residency education had a role in their level of knowledge, so that third year residents compared to the second and first year ones have a higher level of knowledge on dental trauma. Many studies have examined the level of knowledge about dental trauma among healthcare providers. Sood et al. evaluated the physicians' knowledge of emergency management of patients with dental trauma. The results indicate that physicians' knowledge on replantation was 46%, and 60% of physicians were aware of the time of avulsion to replantation. Furthermore, 51% of physicians were aware of preserving the tooth for replantation, whereas 47% of them had no accurate information about the environment to keep the injured tooth. They concluded that physicians' knowledge of dental trauma was insufficient (27). These results are similar to those of the present study. However, as the type of questionnaires used by Sood et al. and the present study was different, it is impossible to compare the questions in detail.

Another study in United Arab Emirates indicated an inadequate knowledge of emergency department physicians and residents about the management of traumatic dental injuries (28). Also, the results of a study in Turkey showed that the majority of emergency department physicians had low knowledge about the diagnosis and treatment of dentofacial traumatic injuries (29). The results of another study in Turkey revealed a lack of knowledge of emergency medicine physicians and nurses regarding management of dentofacial trauma in pediatric patients (30). Wolfer et al. revealed a generally inadequate knowledge and skills in dental trauma management among German emergency physicians (31). Additionally, Bahammam et al. in Saudi Arabia indicated that the majority of physicians in emergency departments have a lack of knowledge for managing avulsions cases (32). Hashim assessed the knowledge and experience of physicians about the management of avulsed teeth in the United Arab Emirates. The results showed that about 68% of the physicians prefer to visit a dentist immediately. None of them like to splint the teeth before visiting a dentist. When the participants were asked about the contents of the avulsed tooth, 42.4% recommended keeping the tooth in normal saline, only ten respondents (8%) knew that milk was the correct response. Finally, few physicians considered appropriate emergency treatment for missing teeth (33). By comparing the results of their study with the present study, it seems that the physicians in both studies did not have enough knowledge about the management of injured teeth. Against the expectations, the results of a study in Turkey revealed that the general dentists had insufficient knowledge regarding the management of traumatized teeth (34). Moreover, the results of a recently published meta-analysis indicated an insufficient level of knowledge regarding emergency management of traumatic dental injuries in most dental professionals (35).

Pithon et al. evaluate the knowledge of Brazilian primary school teachers about rapid action during dental trauma. The study showed that men were more unaware about these quick actions. Indeed, almost half of the teachers were unaware of these actions (36). Also, the results of another study in Saudi Arabia revealed that parents have inadequate levels of knowledge for managing deciduous and permanent tooth avulsion (37). In a study by Joybell et al. which was conducted on employees of emergency ambulance services in India, it has been shown that their overall knowledge regarding management of traumatic injuries to the teeth was not satisfactory (38).

There are some limitations in the present study that need to be addressed. First, given the cross-sectional nature of our study, no inferences of causality could be drawn. Second, in the present study, emergency physicians who worked in hospitals affiliated to Mazandaran University of Medical Sciences were included. Therefore our finding might not be generalizable to all emergency physicians in the country.

In conclusion the results of this study indicated that the mean level of knowledge of emergency physicians regarding the management of dental trauma was average. Emergency medicine specialists had significantly higher level of knowledge compared to emergency medicine residents and GPs. Moreover, there were no statistically significant relationship between physicians' gender, history of participating in dental trauma courses, and their previous experience of managing patients with dental trauma with their level of knowledge about the management of traumatic dental injuries. Therefore, it seems that more attention is required to pay for education of emergency medicine residents, GPs or medical students about appropriate management of traumatic dental injuries.
